# HIV-TB Coinfection among 57 Million Pregnant Women, Obstetric Complications, Alcohol Use, Drug Abuse, and Depression

**DOI:** 10.1155/2018/5896901

**Published:** 2018-01-01

**Authors:** Dorian Fernandez, Imoleayo Salami, Janelle Davis, Florence Mbah, Aisha Kazeem, Abreah Ash, Justin Babino, Laquiesha Carter, Jason L. Salemi, Kiara K. Spooner, Omonike A. Olaleye, Hamisu M. Salihu

**Affiliations:** ^1^Texas Southern University, 3100 Cleburne Street, Houston, TX 77004, USA; ^2^Department of Family & Community Medicine, Baylor College of Medicine, 3701 Kirby Drive, Suite 600, Houston, TX 77098, USA

## Abstract

**Objective:**

HIV and tuberculosis represent diseases of major public health importance worldwide. Very little is known about HIV-TB coinfection among pregnant women, especially from industrialized settings. In this study, we examined the association between TB, HIV, and HIV-TB coinfection among pregnant mothers and obstetric complications, alcohol use, drug abuse, and depression.

**Method:**

We examined inpatient hospital discharges in the United States from January 1, 2002, through December 31, 2014. We employed multivariable survey logistic regression to generate adjusted estimates for the association between infection status and study outcomes.

**Results:**

We analyzed approximately 57 million records of pregnant women and their delivery information. HIV-TB coinfection was associated with the highest risks for several obstetric complications, alcohol use, and drug abuse. The risk for alcohol abuse was more than twice as high among HIV-monoinfected as compared to TB-monoinfected mothers. That risk gap more than doubled with HIV-TB coinfection. Both HIV-monoinfected and HIV-TB coinfected mothers experienced similarly increased risks for depression.

**Conclusions:**

Mothers with HIV-TB coinfection experienced relatively heightened risks for obstetric complications, alcohol use, and drug abuse. The findings of this study underscore the importance of augmenting and enhancing social and structural support systems for HIV-TB coinfected pregnant women.

## 1. Introduction

Human Immunodeficiency Virus (HIV) and tuberculosis (TB) disease represent health issues of major public importance worldwide. Approximately one in three individuals is infected with* Mycobacterium tuberculosis globally*, and HIV is the main risk factor of active tuberculosis disease, increasing the risk of latent TB reactivation 20-fold [1]. Tuberculosis kills more than 1 million women per year, and it is estimated that 646 million women and girls are already infected with tuberculosis [2]. Among women aged 15–44 years in developing countries, tuberculosis is the third most common cause of morbidity and mortality combined and kills more women than any other infectious disease, including malaria and AIDS [3]. HIV-TB coinfected individuals are at greater risk for relapse following treatment as well as increased chances of drug resistance to anti-TB drugs [4–8]. Even in industrialized settings with good healthcare access and antiretroviral therapy (ART) availability, HIV-TB coinfection is associated with high mortality [9].

A recent study suggests that the rate of TB among pregnant women in the United States (US) is climbing, although the increase appears to be as a result of an upsurge in extrapulmonary disease as well as inclusion of pregnant women with reported history of tuberculosis [10]. This poses a concern regarding the effects of TB disease in HIV-positive mothers. Although HIV and TB among pregnant women have been studied separately, the impact of HIV-TB coinfection among affected mothers remains poorly defined or understood. The aim of this study is to determine the association between TB, HIV, and HIV-TB coinfection with pregnancy complications, alcohol use, drug abuse, and depression using a highly reliable and validated population-based dataset. Drawing from more than 57 million pregnancy-related and delivery admissions, this study is the largest in the world on HIV-TB coinfection given the sample size of the source population.

## 2. Materials and Methods

We used data from the Nationwide Inpatient Sample (NIS) covering the period from January 1, 2002, through December 31, 2014, for this analysis. The NIS dataset is made available by the Healthcare Cost and Utilization Project (HCUP) and currently constitutes the largest all-payer, publicly available inpatient database in the US [11]. To create the sample annually, HCUP employs a two-stage cluster sampling design that first stratifies all nonfederal community hospitals from participating states by five major hospital characteristics: rural/urban location, number of beds, geographic region, teaching status, and ownership. Then, 20% of hospitals from each unique stratum were selected using a systematic random sampling technique [12]. In the second stage, all inpatient discharges from hospitals selected during stage 1 were selected for inclusion in the NIS. Beginning in 2012, the NIS sampling strategy was changed from keeping all discharges from a sample of hospitals to drawing a sample of discharges from all hospitals. The NIS approximates a 20% systematic sample that is representative of the population of all discharges on critical hospital and patient characteristics [12]. To assess the study's primary exposures, we scanned ICD-9-CM codes (the principal diagnosis and up to 24 secondary diagnoses) in each woman's discharge record for an indication of HIV and TB status.

Individual-level sociodemographic and behavioral characteristics were also extracted from the NIS databases. Maternal age in years was classified into three categories: 13–24, 25–34, and 35–49. Self-reported maternal race/ethnicity was first based on ethnicity (Hispanic or non-Hispanic [NH]), and the NH group was further subdivided by race (white, black, or other). Median household income, which served as a proxy for socioeconomic status, was estimated using the patient's zip code and subsequently grouped into quartiles. We classified the primary payer for hospital admission into three categories: government (Medicare/Medicaid), private (commercial carriers, private health maintenance organization [HMOs], and preferred provider organization [PPOs]), and other sources (e.g., self-pay and charity). Due to their strong associations with HIV infection, we also used ICD-9-CM codes to ascertain information on alcohol and drug use during pregnancy. Our definition of alcohol abuse included indications of alcohol-induced mental disorders (291.0–9), alcohol dependence syndrome (303.00–93), nondependent alcohol abuse such as binge drinking (305.00–03), alcoholic cardiomyopathy (425.5), and alcohol that has affected the fetus or newborn via the placenta or breast milk (760.71). Drug abuse included drug-induced mental disorders (291.0–9), drug dependence (304.00–93), nondependent drug abuse (305.20–93), drug dependence complicating pregnancy or childbirth (648.30–34), suspected damage to the fetus from drugs (655.50–53), drugs affecting the fetus or newborn via the placenta or breast milk (760.72-73, 760.75), and poisoning by opiates and related narcotics (965.00–09). We also considered several hospital characteristics including teaching status (teaching versus nonteaching), location (urban versus rural), and US region (Northeast, Midwest, South, or West).

### 2.1. Statistical Analysis

Descriptive statistics including frequencies and percentages were used to describe the distribution of index delivery hospitalizations across patient- and hospital-level characteristics and stratified by exposure groups (HIV and TB negative, HIV monoinfection, TB monoinfection, and HIV-TB coinfection). Since national estimates were desired, all statistical analyses were weighted using an HCUP-provided discharge-level weight that accounts for the NIS's sampling design. Multivariable survey logistic regression was then used to generate adjusted odds ratios (OR) that quantified the magnitude of the association between infection status (i.e., HIV monoinfection, TB monoinfection, and HIV-TB coinfection) and the main outcomes of the study (pregnancy complications, alcohol use, and drug abuse). Statistical analyses were performed with SAS, version 9.4 (SAS Institute, Inc., Cary, NC); we assumed a 5% type I error rate for all hypothesis tests (two-sided). Due to the deidentified, publicly available nature of NIS data, the analyses performed for this study were considered exempt by the Baylor College of Medicine Institutional Review Board. It is pertinent to mention that some of the results of our analyses contained small numbers which must be suppressed in accordance with guidelines set forth by the Healthcare Cost and Utilization Project. The reason is to prevent possible identification of these individuals, and, in such cases, we described the findings in text without displaying the actual values.

## 3. Results

We analyzed a total of 57,393,459 hospitalizations related to pregnancy and delivery over the study period. The diagnosis of active TB monoinfection was documented in 4,053 mothers yielding a rate of 7.06 per 100,000. In the entire population of mothers, the prevalence of HIV monoinfection was 12.76 per 10,000 (*N* = 73,223 HIV-infected mothers). Although the prevalence of HIV-TB coinfection was 1.9 per million (*N* = 110) in the entire population of pregnant mothers, the rate of TB among HIV-positive mothers was 150.23/100,000. This was 21 times as high when compared to the rate of TB in the entire population of pregnant women.

**Figure 1:**
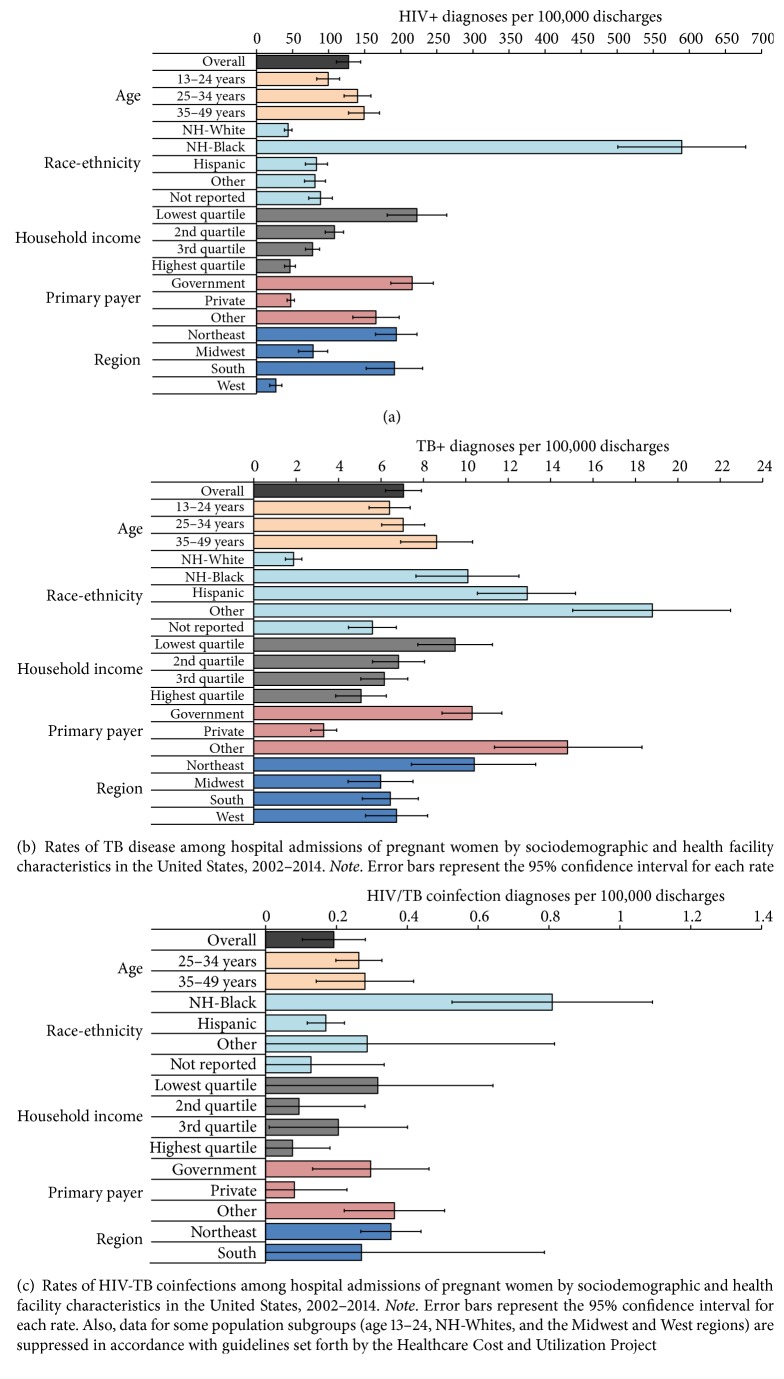


Table 1 and Figures 1(a), 1(b), and 1(c) represent a summary of sociodemographic characteristics by infection status. HIV-TB monoinfection and HIV-TB coinfection correlated positively with ascending age, with notable racial/ethnic differences. The rates of HIV and HIV-TB coinfection were highest among black mothers, while TB monoinfection was most prevalent in Hispanics. Whereas the proportion of HIV monoinfection, TB, and HIV-TB coinfection was 13-, 5-, and 20-fold as high among black mothers when compared to their white counterparts, respectively, the corresponding proportion among Hispanics was 2, 7, and 4 times as high as that of their white counterparts, respectively. Low socioeconomic status and having public or no health insurance were the greatest risks for HIV-TB monoinfection and HIV-TB coinfection. Our data also portrayed geographical differences in the rates of infection among mothers. The Northeast had the highest rates for HIV-TB monoinfection and HIV-TB coinfection, while the Western part of the US had relatively low prevalence of HIV and HIV-TB coinfection. Across health facilities, the rates for HIV-TB monoinfection and HIV-TB coinfection were highest among urban teaching hospitals and lowest for hospital facilities located in rural areas.

Table 2 summarizes the rates of pregnancy complications, alcohol use, drug abuse, and depression among pregnancy-related hospital admissions for the entire period of the study. Note that some of the values in the table are suppressed due to small numbers in accordance with guidelines set forth by the Healthcare Cost and Utilization Project. The reason is to prevent possible identification of these individuals, and, in such cases, we described the findings in text without displaying the actual values. Pregnancy complications as a composite outcome were highest among HIV-TB coinfected mothers followed by those with TB monoinfection. Since we only observed 110 cases of HIV-TB coinfection out of the more than 57 million hospital admissions, it was to be expected that not all pregnancy complications would occur in the 110 HIV-TB coinfected mothers. Four (eclampsia, placenta accreta, postpartum hemorrhage, and anemia) of the 10 pregnancy complications were detected among women with HIV-TB coinfections. When the rates of these complications were compared across the categories of infection status, three of the four complications (eclampsia, placenta accreta, and anemia) occurred with greatest frequency among HIV-TB coinfected mothers. The only exception (postpartum hemorrhage) was highest among women with TB monoinfection. We then compared the other remaining six complications that had zero occurrence among HIV-TB coinfected mothers in disease-free (no TB and no HIV), HIV-monoinfected, and TB-monoinfected mothers. We found similar rates of preeclampsia across the three groups. Diabetes mellitus, placental abruption, and other sources of antepartum hemorrhages were highest among HIV-monoinfected mothers while placenta previa and sepsis were greatest among mothers with TB monoinfection. Alcohol abuse and drug abuse rates were consistently greatest among mothers with HIV-TB coinfection followed by those with HIV monoinfection while the prevalence of depression was most pronounced among HIV-monoinfected mothers.

Table 3 describes the association between infection status and obstetrics complications, alcohol abuse, drug abuse, and mental health. The risk for pregnancy complications, even after adjustment for alcohol use, drug abuse, and depression, was greatest among women with HIV-TB coinfection [OR = 2.00 (0.83, 4.79)] although this 2-fold elevated risk level did not reach statistical significance. Mothers with HIV monoinfection exhibited the lowest likelihood for pregnancy complications [OR = 1.40 (1.32, 1.47)]. We did observe moderation of the effect of HIV/TB status on pregnancy complications by substance abuse behaviors and depression. Whereas women without any indication of alcohol use, drug abuse, or depression had associations between HIV/TB status and pregnancy complications that were similar to the entire study population, measures of association were greatly attenuated among women with substance abuse or depression (data not shown). The likelihood for alcohol abuse was more than twice as high among HIV-positive patients [OR = 5.12 (4.42, 5.93)] as compared to TB-monoinfected patients [OR = 2.10 (0.79, 5.55)]. That risk gap more than doubled with HIV-TB coinfection [OR = 11.39 (1.38, 94.06)]. Mothers with HIV or HIV-TB coinfection experienced elevated risks for drug abuse and depression almost twice as much as those with TB monoinfection only.

## 4. Discussion

The prevalence of 1.9 HIV-TB coinfection cases per one million pregnant women in our study is lower than that for resource-limited nations, although our denominator (more than 57 million admissions) is the largest reported. It is noteworthy that the rate of TB among HIV-positive mothers was 150.23/100,000 in the population we analyzed, which was 21 times as high when compared to the rate of TB in the entire population of pregnant women analyzed. This demonstrates the extremely high vulnerability of HIV-infected persons to TB disease. People infected with HIV are at high risk for developing active TB due to either the reactivation of a latent infection or the progression of newly acquired tubercle bacilli infections. Among HIV-positive individuals, the immune responses to tubercle bacilli may not be effective due to underlying HIV-induced immunological aberrations which weaken effector immune responses [13, 14]. Results from immune phenotyping studies indicate that peripheral CD4+T cells from a subset of HIV-positive patients on ART regimens still continue to manifest characteristic features of pronounced cellular exhaustion [15], turnover [16], and senescence [17]. These aberrant immunological patterns will explain the raised likelihood of tuberculosis disease and reactivation of latent tuberculosis in HIV-positive pregnant mothers as reported in this study.

We also found HIV-TB coinfection to be associated with the greatest risk for pregnancy complications as compared to HIV and TB monoinfection status. However, the almost 2-fold elevated risk among HIV-TB coinfected mothers had a wide confidence interval that encompassed the null. This might be due to the small number of cases. Nonetheless, our results are in congruence with previous studies in low-resource high-HIV prevalence settings which demonstrated significant maternal morbidity and mortality in pregnant women with HIV-TB coinfection [18–20]. In a recent analysis by Canadian authors utilizing the same dataset covering the period 2003 to 2011, the investigators reported elevated risks of chorioamnionitis, preterm labor, postpartum anemia, blood transfusion, and pneumonia as well as maternal mortality among pregnant women with tuberculosis disease [10]. Our study showed that HIV-coinfected women were particularly at high risk for eclampsia, placenta accreta, and anemia. The heightened risks of maternal complications among mothers with HIV-TB coinfection represent a pathway for the increased risk of maternal mortality consistently reported among HIV-positive mothers with TB coinfection.

We also observed elevated risk for alcohol use which was more than twice as high among HIV-positive patients [OR = 5.12 (4.42, 5.93)] as compared to TB-monoinfected patients [OR = 2.10 (0.79, 5.55)]. That risk gap more than doubled with HIV-TB coinfection [OR = 11.39 (1.38, 94.06)]. Mothers with HIV or HIV-TB coinfection experienced elevated risks for drug abuse and depression, almost twice as much as those with TB monoinfection only. A potential mechanism that explains the strong positive association between HIV positivity and excessive alcohol use, drug abuse, and depression is perceived stress [21]. HIV-positive persons who experience the greatest stress in their daily lives are those who disengage behaviorally/emotionally in coping with their illness and those who approach their interpersonal relationships in a less secure or more anxious style [22]. By contrast, studies indicate that, for people with HIV or AIDS, those individuals who are more satisfied with their relationships, securely engaged with others, and more directly engaged with their illness are more likely to experience positive adjustment [23–25]. It is noteworthy to mention that reverse causality does exist in the relationships between HIV on one hand and alcohol use/drug abuse on the other, since drug abusers and alcohol users tend to engage in behaviors that put them at elevated risk for HIV infection [26, 27]. The nature of social support could also influence the likelihood of risk behaviors among HIV-positive individuals. Previous investigators have noted the importance of the lack of positive social support or the existence of “negative” support as an important factor that encourages sexual and drug-related HIV risk behavior such that illegal drug use by members of a social network often provides peer support for continued risk behavior [28]. Our results tend to suggest that the concomitant existence of TB disease among HIV-positive individuals increased the risk for alcohol use and drug abuse although the magnitude of the excess risk was more pronounced for alcohol use.

The results of our analysis underscore the importance of augmenting and enhancing social and structural support systems for HIV-TB coinfected pregnant women because of the increased risk for pregnancy complications in addition to the associated raised likelihood of alcohol use and drug abuse. Given the significant findings in this study, tailored interventions are needed to maximize the effectiveness of management modalities of HIV-TB coinfected pregnant mothers in order to prevent or mitigate the health consequences of concomitant alcohol use, drug abuse, and depression. It is recommended that HIV-TB coinfected mothers be supported with sufficient provider-patient communication and contact time before discharge in order to reinforce understanding of their condition and link them with community assets to cater for their future needs and care related to sustained pregnancy complications.

Even though the dataset used for this study is nationally representative and rich with an array of clinical and epidemiologic measures, certain limitations merit mentioning. It is logical to assume that the quality of care received for the management of HIV and TB among the mothers in this study would vary considerably across the diverse population settings in the United States. We lack a marker/measure of quality of care or adherence with HIV care protocols in the dataset to assess variation in quality of management of HIV and TB conditions which could have influenced the occurrence of the maternal pregnancy complications examined in this study. It is notable that 60% of the pregnancy complications were not detected among women with HIV-TB coinfection due to the rarity of HIV-TB coinfection (1.9 cases per million). Another shortcoming in this study is the lack of biological markers (e.g., CD4 cell count and HIV load) to predict mothers that were likely to develop pregnancy complications, or to determine the association of these biologic markers with alcohol use, drug abuse, or depression. Despite these limitations, our analysis is based on the most powerful data so far in terms of sample size (57 million) for the source population of HIV-TB coinfected pregnant women and the range of outcomes examined in our study.

## Figures and Tables

**Table 1:** Distribution of sociodemographic and health facility characteristics among hospital admissions of pregnant women, by HIV/TB status, United States, 2002–2014.

	HIV/TB status			
	HIV–, TB− *n* = 57.3 m^a^	HIV+, TB− *n* = 73,223	TB+, HIV− *n* = 4053	HIV+, TB+ *n* = 110
	%^b^	%^b^	%^b^	%^b^
*Age*				
13–24	33.9	26.4	31.3	c
25–34	51.4	56.5	50.8	70.3
35–49	14.7	17.2	17.8	21.4
*Race/ethnicity*				
NH-White	42.1	14.5	11.3	c
NH-Black	11.8	54.8	16.0	49.9
Hispanic	18.9	12.3	34.8	16.7
Other	8.6	5.4	23.0	12.7
Not reported	18.7	13.0	14.9	12.4
*Income*				
Lowest	27.1	47.3	36.3	44.7
2nd	24.8	21.0	24.3	12.1
3rd	24.1	14.6	20.9	25.7
Highest	22.2	8.1	16.1	8.7
*Payer*				
Government	43.0	72.7	62.6	66.4
Private	50.5	18.8	23.6	21.3
Other	6.5	8.5	13.8	12.3
*Hospital region*				
Northeast	16.5	25.1	24.0	30.3
Midwest	21.2	13.1	18.3	c
South	37.9	56.8	34.0	53.2
West	24.4	5.1	23.7	c
*Hospital type*				
Rural	11.2	3.3	4.8	c
Urban, nonteaching	40.4	16.7	20.0	16.4
Urban, teaching	48.0	79.5	74.6	79.3

^a^The estimated number of records with HIV–, TB– is 57,316,293. ^b^Displayed percentages are column percentages. Percentages may not add to 100% due to missing data. ^c^Data are suppressed due to small numbers in accordance with guidelines set forth by the Healthcare Cost and Utilization Project.

**Table 2:** Rates (per 1,000 hospitalizations) of alcohol use, drug abuse, depression, and complications of pregnancy among pregnancy-related admissions, by HIV/TB status, United States, 2002–2014.

	HIV/TB status			
	HIV−, TB− *n* = 57.3 m^a^	HIV+, TB− *n* = 73,223	TB+, HIV− *n* = 4053	HIV+, TB+ *n* = 110
Alcohol use	1.7	16.1	4.7	b
Drug abuse	16.0	114.6	25.3	119.08
Depression	20.1	53.2	25.8	b
Pregnancy complications (all)	166.4	272.2	292.1	331.28
Anemia	102.7	214.1	216.6	206.33
Diabetes	11.3	24.6	16.7	b
Preeclampsia	23.4	23.8	24.0	b
Eclampsia	1.0	1.4	2.8	b
Placental abruption	10.6	11.6	8.9	b
Placenta previa	5.2	5.5	9.9	b
Placenta accreta	3.3	2.6	1.3	b
Others^c^	3.6	4.9	3.6	b
Postpartum hemorrhage	25.8	21.6	45.5	b
Sepsis	1.3	5.4	8.3	b

^a^The estimated number of records with HIV−, TB− is 57,316,293. ^b^Data are suppressed in accordance with guidelines set forth by the Healthcare Cost and Utilization Project. ^c^Other antepartum hemorrhages.

**Table 3:** Adjusted odds ratios for the association between HIV infection, TB disease, and HIV-TB coinfection status and obstetrics complications, alcohol abuse, drug abuse, and mental health among pregnancy-related admissions in the Unites States.

Status	Outcome			
	Pregnancy complications	Alcohol use	Drug abuse	Depression
HIV−, TB−	1.00 (reference)	1.00 (reference)	1.00 (reference)	1.00 (reference)
HIV+, TB−	**1.40 (1.32, 1.47)**	**5.12 (4.42, 5.93)**	**4.63 (4.19, 5.12)**	**2.83 (2.60, 2.39)**
TB+, HIV−	**1.91 (1.64, 2.23)**	2.10 (0.79, 5.55)	1.39 (0.92, 2.12)	1.54 (1.00, 2.39)
HIV+, TB+	2.00 (0.83, 4.79)	**11.39 (1.38, 94.06)**	**5.08 (1.43, 18.04)**	2.55 (0.34, 19.09)

*Note*. Statistically significant adjusted odds ratios (95% confidence interval does not include 1) appear in bold text. Models for all outcomes are adjusted for age, race, income, payer, year of hospitalization, and hospital region; the model with pregnancy complications as the outcome is also adjusted for alcohol use, drug abuse, and depression.
